# Optimized CapsNet for Traffic Jam Speed Prediction Using Mobile Sensor Data under Urban Swarming Transportation

**DOI:** 10.3390/s19235277

**Published:** 2019-11-29

**Authors:** Hendrik Tampubolon, Chao-Lung Yang, Arnold Samuel Chan, Hendri Sutrisno, Kai-Lung Hua

**Affiliations:** 1 Department of Computer Science and Information Engineering, National Taiwan University of Science and Technology, Taipei 106, Taiwan; D10715806@mail.ntust.edu.tw (H.T.); hua@mail.ntust.edu.tw (K.-L.H.); 2 Department of Industrial Management, National Taiwan University of Science and Technology, Taipei 106, Taiwan; M10601803@mail.ntust.edu.tw (A.S.C.); D10701802@mail.ntust.edu.tw (H.S.)

**Keywords:** traffic jam prediction, urban swarming transportation, capsule network, convolution neural network, smart city

## Abstract

Urban swarming transportation (UST) is a type of road transportation where multiple types of vehicles such as cars, buses, trucks, motorcycles, and bicycles, as well as pedestrians are allowed and mixed together on the roads. Predicting the traffic jam speed under UST is very different and difficult from the single road network traffic prediction which has been commonly studied in the intelligent traffic system (ITS) research. In this research, the road network wide (RNW) traffic prediction which predicts traffic jam speeds of multiple roads at once by utilizing citizens’ mobile GPS sensor records is proposed to better predict traffic jam under UST. In order to conduct the RNW traffic prediction, a specific data preprocessing is needed to convert traffic data into an image representing spatial-temporal relationships among RNW. In addition, a revised capsule network (CapsNet), named OCapsNet, which utilizes nonlinearity functions in the first two convolution layers and the modified dynamic routing to optimize the performance of CapsNet, is proposed. The experiments were conducted using real-world urban road traffic data of Jakarta to evaluate the performance. The results show that OCapsNet has better performance than Convolution Neural Network (CNN) and original CapsNet with better accuracy and precision.

## 1. Introduction

The term “smart city” has been defined by IBM [1] to indicate a smart city that utilizes information and communication technology to analyze and integrate the data into core systems for running the city. The key enabler of the smart city depends on the connected devices and how the collected data, generated through the Internet of Things (IoT) sensors [2], is used. As the volume and variety of data offered by the IoT keeps increasing exponentially, how to utilize the data and transform it to knowledge for a smart city are crucial tasks for modern civilization.

A large amount of data collected from speed sensors or surveillance camera systems have been used to monitor traffic conditions on roads in an intelligence traffic system (ITS) domain. The most common detection technologies are loop detector, road-side cameras, and on-board equipment [3]. Lv, Yisheng, et al. utilized California’s freeway traffic detector station data to predict traffic flow [4]. Zhao, Chen also utilized Beijing’s ring road observation station, which is equipped with cameras, induction coils, and velocity radars to make short-term forecasts of traffic volumes [5]. Similarly, closed circuit television camera (CCTV), laser detector, and loop detector data were utilized by Kim and Hong to predict the traffic flow in Suwon city intersection roads in South Korea [6]. All of the mentioned research works relied on data from fixed traffic detectors which are costly infrastructures of a transportation system. Therefore, how to obtain less costly and more accurate traffic flow information is an important issue in this research area.

Limited budgets for ITS implementation in developing countries where the transportation infrastructure is relatively straggly hinders the detection of traffic conditions. Fortunately, the telecommunication infrastructure in these developing counties comparatively prevails. The use of smartphones, in fact, provides a sufficient source of traffic data by tracing the global positioning system (GPS) information. For example, the government of Jakarta city, the capital city of Indonesia, has taken advantage of the data feed of citizen’s smartphones to aggregate the data for traffic usage by collaborating with Waze® [7], which is a popular navigation software installed on citizens’ smartphone. Instead of having static positions of vehicles, the location information is dynamic, following the smartphone’s location. Because the volume of signals from smartphones is huge, data preprocessing is needed to generate some indication of traffic jam information. 

Currently, traffic prediction has been developed to deal with challenges and concerns as summarized in [8]. With respect to an urban road network, as mentioned in [8], the main challenges that traffic prediction faces include: (1) A more complex road network (large scale) should be considered due to the urban environment, (2) the model should include spatial-temporal (ST) characteristics of the traffic network to describe the corresponding conditions among multiple roads, and (3) utilization of artificial intelligence is still an emerging research area. How to specifically develop a deep learning framework in this area is an important research topic.

Understanding, monitoring, and predicting traffic road conditions, such as the level of traffic jam at certain times, are essential needs of urban transportation [9]. Over the past decade, there have been many studies conducted on the traffic prediction model. Most of the existing models have employed statistical, time series based, probabilistic, and neural network approaches according to the reviews [8,10,11]. The following models (1) time series analysis model, (2) traditional machine learning, and (3) deep learning based model, are summarized below.

(1) Time Series Analysis Model

Time series analysis approaches such as ARIMA [12] and sliding window ARIMA (SWARIMA) [9] have been used to study the traffic conditions for the purpose of predictions. Traffic flow data recorded from sensors are frequently noisy, and the short-term traffic prediction is considered as a nonstationary. To deal with this issue, Xie et al. applied a Kalman filter (KL) to handle nonstationary for short-term prediction [13]. In their work, wavelet decomposition analysis was utilized to reduce the noise. Similarly, their model was applied to a relatively simple freeway road network. Yan et al. also proposed a traffic flow prediction based on multivariate time series to understand the traffic patterns on the freeway [14]. In their work on traffic volume, occupancy, and speed, multivariate traffic time series were utilized and converted into a complex network structure. 

Most of these approaches mainly focus on the following: (1) predicting traffic flow at a single data point, (2) considering traffic data as a sequence, and (3) finding the patterns of the temporal variation of traffic on one road segment. However, the road traffic condition actually can be propagated among the road network as mentioned in [15]. This means that if one road is badly jammed, the traffic is propagated to other nearby roads which causes the network effect. Because of this propagation characteristic, the road network wide (RNW) traffic prediction which predicts traffic conditions of multiple roads at once is needed, not only based on the time series data analysis, but also the network structure of roads. 

(2) Traditional Machine Learning Model

In addition to the traditional time series analysis, machine learning models such as support vector machine (SVM), neural network (NN), and deep learning (DL) network have been applied to the traffic prediction domain for decades. Tang et al. proposed a fuzzy neural network model to predict traffic speed by considering periodic characteristics [16]. In their work, k-means was employed to extract periodic features of travel speed data that had been collected from three adjacent stations. Then, a trigonometric regression was used to predict travel speed for multi-step ahead. Although the proposed model performed well on single traffic road prediction, the model did not consider road network perspective, as mentioned before.

Many SVM-based models have been widely employed for traffic prediction. For example, Zeng et al. proposed AOSVR to deal with time efficiency of the traffic flow prediction [17], Saldana-Perez et al. took advantage of social media data to characterize the traffic congestion and analyze crowd-sensed data from a geospatial perspective [18]. Yan, H. and D.-J. Yu. proposed an improved SVM to classify traffic jam conditions which was able to handle a negative effect of an outlier on the traffic data [19]. In their study, relatively small data were used, and therefore their model failed to deal with large-scale traffic prediction.

Furthermore, Tang et al. proposed a hybrid model of SVM with several denoising techniques to predict traffic volume at multiple ahead steps (2 min, 10 min, and 60 min time horizon) [20]. Empirical mode decomposition (EMD), ensemble empirical mode decomposition (EEMD), moving average (MA), Butterworth (BW) filter, and wavelet (WL) were combined with SVM. According to their experiments, obviously, EEMD outperformed as compared with other denoising techniques. Although the proposed model performed well to predict multiple ahead steps traffic conditions, the spatial correlation between the detectors was not considered in their study. Moreover, the predictive model only focused on the freeway and not the urban environment. 

(3) Deep Learning Based Model

Recently, more advanced approaches such as deep learning model have been applied to traffic prediction due to their promising performance. For example, Kim et al. utilized the much deeper and complex recurrent neural network (RNN) model to predict traffic speed [21]. Abbas et al. used long short-term memory (LSTM) model, a type of RNN, for the short-term traffic prediction on the road [22]. In addition, LSTM has also been used for travel time prediction [23]. The RNN and LSTM models are state-of-the-art and powerful for capturing temporal features in traffic. However, the spatial interaction is not considered from the point of view of the road network, although the forecasting task is on a single road in a relatively small region.

Realizing local dependencies of a network can improve the prediction. Song et al.’s work predicted Seoul’s main road traffic speed on weekdays utilizing CNN and obtained better results than two multilayered perceptron network [24]. Another research took advantages of both LSTM and CNN to capture a spatial-temporal correlation to predict travel time [25]. Nevertheless, in the literature, few studies have considered the RNW as a whole to exploit the correlation of spatial-temporal features effectively and estimate the traffic interactions among the road segments on a large scale. Especially, for urban roads, knowing the traffic condition in a whole road network, instead of a single road, can result in better decision making by transporters.

To address this concern, Ma et al. proposed a novel approach to learn traffic data as image and applied CNN to predict traffic speed on roads network wide instead of single road segment [26]. The model was evaluated on Beijing’s ring road network using traffic data from taxis’ GPS across the city and achieved 42% accuracy improvement as compared with other algorithms such as KNN, ANN, random forest, and least square methods. In addition, in studies by [27,28], CNN-based model was also compared to traditional ANN. Their findings confirmed that deep learning based model outperforms the traditional network (ANN), other machine learning methods, and statistical methods.

As evident in the literature, CNN has a drawback in the pooling operation which was addressed in Sabour et al.’s work [29]. To tackle the limitation of CNN, Sabour et al. introduced a new type of neural network, called capsule network (CapsNet). A capsule is a group of neurons which has different properties from the same entity. It is trained by dynamic routing instead of max-pooling and has a different nonlinearity, namely squash. Since then, many research works applied CapsNet to train the prediction model, including the traffic prediction problems. Extending what Ma et al. has done, Kim et al., applied CapsNet to the traffic speed prediction problem [30]. In their work, the max-pooling operator inside CNN was found to lead to information loss of the interaction among road characteristics in urban transportation. Therefore, replacing the max-pooling operator with routing by agreement algorithm in CapsNet improved the prediction result.

**Figure 1:**
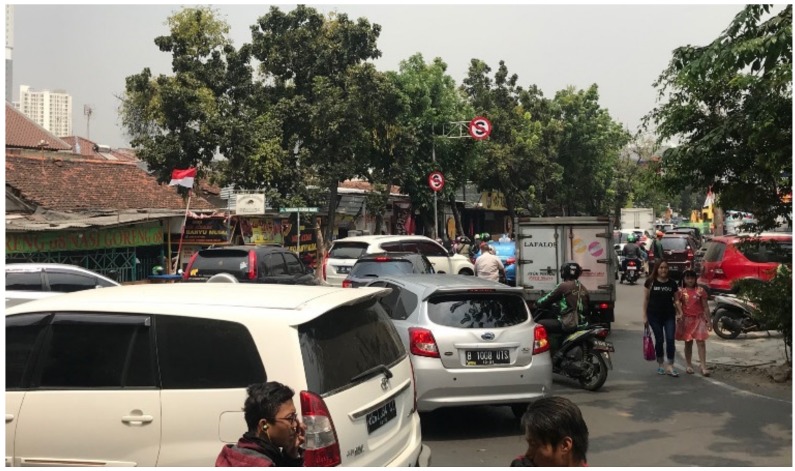
An example of urban swarming transportation” (UST) condition (pictures taken by authors on 24 August 2019, in Jl. Tanjung Duren Raya, West Jakarta, Indonesia).

In this study, we develop a prediction model focusing on predicting traffic jam speed on urban roads based on the information collected from citizens’ smartphones. In order to differentiate the urban roads where this work focused from the free-flow and well-regulated road transportation, we introduce the term “urban swarming transportation” (UST). Essentially, in UST, the lane marks on most of the UST are not clear or even not existing. It means that all kinds of vehicles, pedestrians, and even animals share the same road. The traffic light system on UST in some developing countries is insufficient or not strictly followed by transporters. Figure 1 shows a typical example of the UST condition (the photo was taken in west Jakarta). As shown in Figure 1, the UST roads are swarmed with all kinds of the mentioned transporters. Note that the road in the picture is not “one-way” and all transporters can use this bidirectional and narrow road.

In order to handle the traffic data collected from mobile phones, data preprocessing is needed to map the traffic speed data originally with longitude and latitude, to the traffic measurement with the road sections. In this study, two deep learning methods, CNN and its extension, CapsNet, are used to train the prediction model to predict the traffic jam speed on the UST roads. Note that the CNN model is the benchmark of our work for comparison purposes. In addition, optimized CapsNet (named OCapsNet) architectures is proposed to change the ReLU nonlinearity at the first two layers of the convolution step. Edgar Squash is applied to the capsule layer in the original CapsNet. We propose the use of some strategies to tweak dynamic routing as mentioned in [31,32,33], and therefore obtain better prediction performance.

The contributions of this paper are summarized as follows:

- We used traffic data recorded by mobile sensors such as GPS, instead of fixed detectors on the road, as a cost efficiency for traffic prediction on urban roads under UST;
- We proposed CapsNet-based traffic jam prediction as a comparable to CNN-based predictive model to deal with the RNW condition which is the complex road network and spatial-temporal traffic road characteristics under UST;
- We improved the performance of CapsNet by utilizing nonlinearity function in the convolution layer of CapsNet to modify dynamic routing on the two-capsule layer of the original CapsNet.

This paper is organized as follows: Section 2 addresses the techniques used for traffic data preprocessing. Section 3 describes the details of traffic jam speed prediction tools. Section 4 outlines the experimental setup in deep learning methods and the experimental results. Finally, the conclusion and future study directions are mentioned in Section 5.

## 2. Traffic Data Preprocessing

In this research, one year of traffic jam speed data on urban roads in the Jakarta metropolitan has been used as a sample data of UST for studying. The data is collected by the governmental independent smart city division of Jakarta named Jakarta Smart City (JSC). The traffic speed in any Jakarta area is captured in every second interval from Waze mobile app. The measurement of 15 min and 5 min are used to aggregate the traffic jam records. According to information described by JSC, traffic speeds higher than 10 km/h can be considered as free flow (the traffic in Jakarta is extremely congested). Therefore, the collected raw dataset only keeps the traffic jam records. It should be noted that five traffic jam levels are associated with the traffic jam speed (lower than 10 km/h) of each record which comes with longitude and latitude position. Table 1 shows examples of traffic jam speed data. The traffic speed associated with each traffic jam level is categorized as follows:

- Level 0 interpreted as free flow (not recorded);
- Level 1 is 6.1 km/h to 8.1 km/h of traffic speed;
- Level 2 is 4.1 km/h to 6.1 km/h of traffic speed;
- Level 3 is 2.1 km/h to 4.1 km/h of traffic speed;
- Level 4 is bigger than 0.0 km/h to 2.1 km/h of traffic speed;
- Level 5 is 0.0 km/h which is denoted as blocked.

Four relevant attributes from the urban traffic jam data were chosen in this research. They are time occurrence, traffic speed, longitude, and latitude of the traffic jam, as shown in Table 1. In order to identify the traffic jam location on a certain urban road, the external road information offered by a public traffic road database (OSM) is used [34]. 

**Figure 2:**
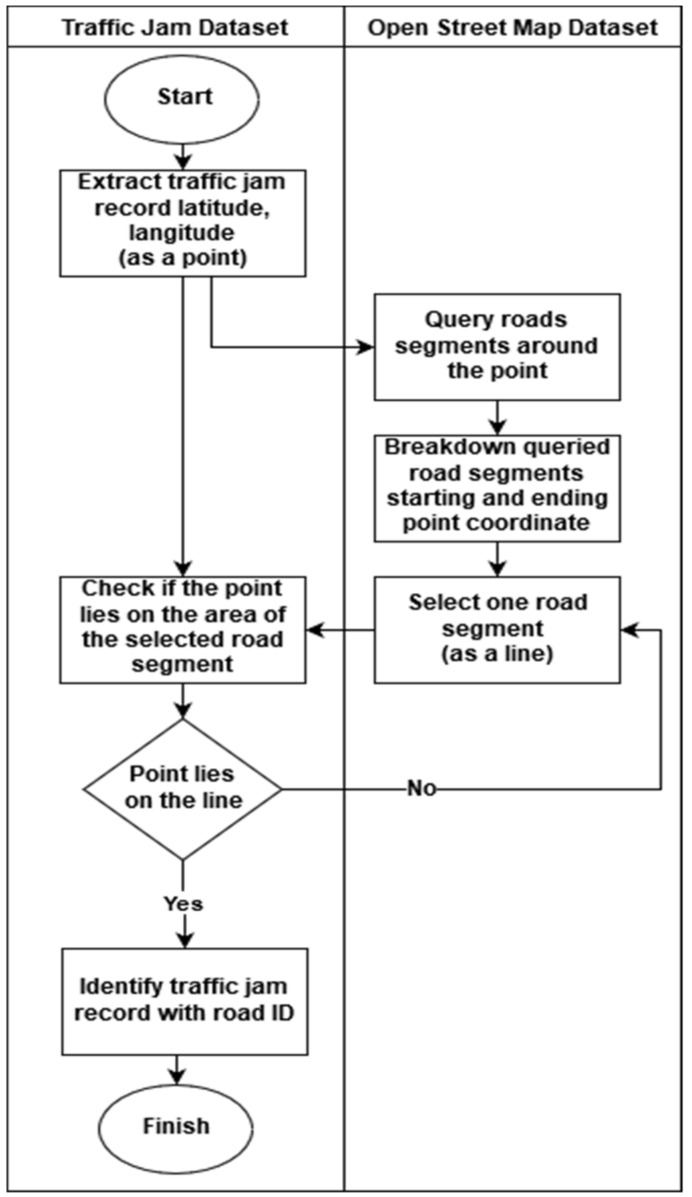
The integration process of the traffic jam dataset and OSM dataset.

The process of integrating datasets of traffic jam dataset (Table 1) and OSM is shown in Figure 2. The first step is to extract the coordinate information (latitude and longitude), and traffic jam measure from the dataset as a data point. Secondly, on the OSM, querying out 10 (or more, by setting) road segments which are near to the data point, and connecting the starting and ending coordinates of each found road segment as a line. Third, assuming each road centered with a line has a certain width, check if the point lies on the road. If yes, the traffic measure of the point can be associated with the found road segment. If no, then keep checking if the point can be associated with the other road segment. If one point does not lie on all found road segments, it means the coordinate of the point is too far from the road and can be considered as a useless point.

Every road section in the OSM database can be identified with an OSM ID. A single road with a road name may have multiple OSM IDs to represent road sections if the road is very long. Every road section can be broken into smaller road segments identified as Road ID. Adding OSM ID and Road ID, the traffic jam record’s coordinates (longitude and latitude) can be located at the same road segment’s nearby. Table 2 shows the example of the integrated traffic data with OSM.

In order to emphasize the research problem, eight main roads in the JSC dataset are chosen, as listed in Table 3. These roads located in the central Jakarta City were used to represent typical Jakarta’s urban traffic road with extremely jam-packed traffic conditions. It is noted that multiple kinds of vehicles and pedestrian are allowed to commute on the chosen roads which show a typical representation of UST traffic condition roads existing in most developing countries. Examples of the road sections (OSM ID) and the associated road segments (Road ID) are shown in Table 4.

**Figure 3:**
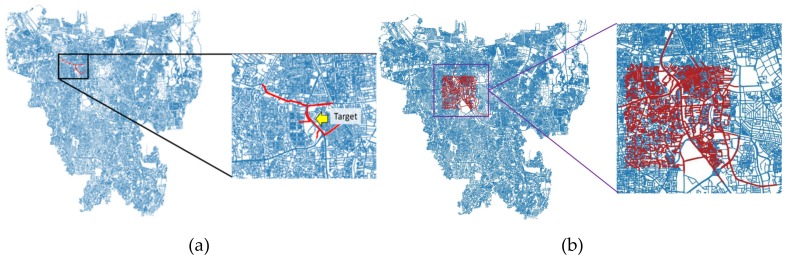
Open street map Jakarta’s urban traffic road visualization: (**a**) Entire Jakarta’s urban traffic road map (left) and the chosen Jakarta’s urban traffic 61 road segment on 8 main roads (right) and (**b**) entire Jakarta’s urban traffic road map (left) and the chosen more complex Jakarta’s urban traffic road 5 × 5 km square area (right).

In this study, a prediction model was developed to predict traffic flow on the selected road segments which presents typical UST road conditions, as shown in Figure 3, while the information of supplementary road segments is used as additional feed to the prediction. These supplementary roads are chosen by considering target adjacency and representing the main road where spatially correlated with each other. In this study, 2,131,584 rows of fifteen-minute interval data and 6,401,890 rows of five-minute interval data were used. Using these interval data, two datasets are prepared for the experiments. One smaller dataset contained 61 distinct road segments (Road ID) that represented eight distinct selected roads, as shown in Figure 3a. Another larger dataset contained 2972 road segments on 5 × 5 km coverage area, as shown in Figure 3b. Examples of data features used in this work are shown in Table 5.

## 3. Traffic Jam Speed Prediction Model

In this study, we aim to propose a traffic jam prediction model that is based on the following considerations: (1) traffic data recorded by mobile sensors such as GPS were used, instead of using fixed detectors on the road, (2) spatial-temporal characteristics of data were used as input image, (3) RNW prediction was conducted to capture the traffic conditions on the entire road network, instead of on a single road, and (4) the CapsNet-based model as an extension of CNN was employed to understand the ST traffic characteristic under UST. In order to achieve the motivation aforementioned above, the traffic jam data are first transformed as an image which contains the traffic jam information of multiple time steps, and feds into the model. The detailed approaches are introduced in the following section.

### 3.1. Converting Traffic Jam Speed as an Image

**Figure 4:**
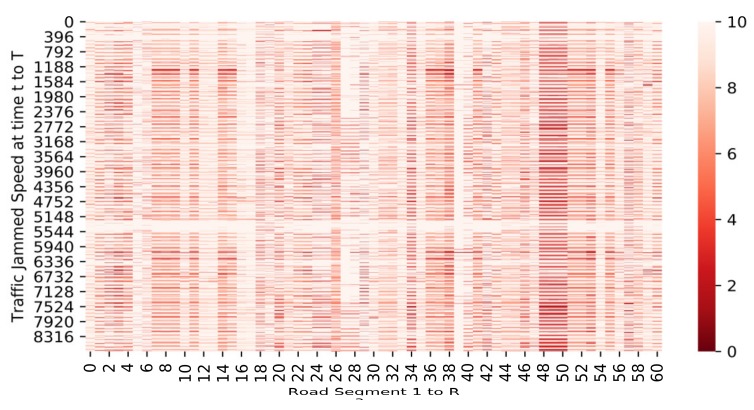
The input of the network is considered as the images T × R, R is the total road segment, and T is time horizon representing the number of 5 min intervals, and t represents every five min traffic jam speed.

In order to fit the traffic data to the CapsNet network, we then consider the recorded traffic jam speed as a pixel of the one-channel image with R-dimension. It also means a T x R matrix should be constructed as denoted in Equation (1), where R is the number of total road segments and T is time horizon representing the number of 5 min intervals. Assuming that VS is the input space passing to the CapsNet network, the output VSOUT can be denoted in Equation (2) as an output matrix with L × R dimension, where L is the number of time horizon to be predicted. Figure 4 illustrates one example of converting traffic jam data into image.

(1) $$VS=\left[\begin{array}{ccc} VS_{11\;} & \cdots & VS_{1R\;}\\ \vdots & \ddots & \vdots \\ VS_{T1\;} & \cdots & VS_{TR\;} \end{array}\right],$$

*VSOUT* = [*VOUT*_1_ … *VOUT_R_*, *VOUT*_*R*+1_ … *VOUT_LR_*]. (2)

### 3.2. CNN-Based Traffic Jam Prediction

In this research, CapsNet was the proposed method and CNN model was considered as a benchmark to show how the traffic jam speed predictive model as an image perspective can be achieved. To reduce the confusion, we skip the introduction of CNN. Detailed information about CNN can be found in [35]. Here, we only address how the CNN network can be used to deal with traffic jam speed prediction under UST.

**Figure 5:**
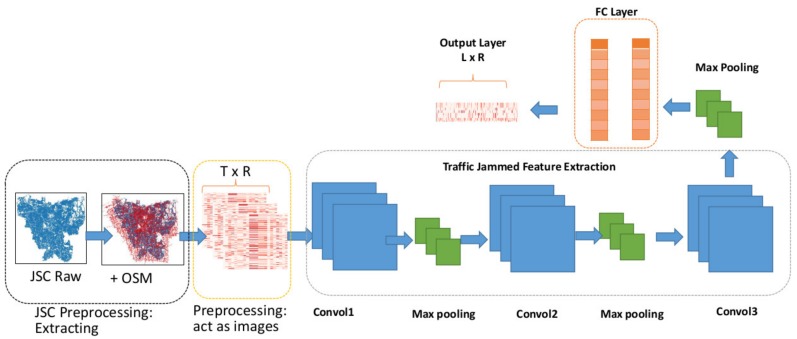
CNN-based traffic jam prediction framework [26].

Figure 5 depicts the CNN network structure followed by [26] which was used as a benchmark for comparison. Note that the research work in [26] predicted traffic speed in Beijing ring road, but our work not only predicted the traffic jam speeds which are relatively much slower, but also focused on RNW traffic prediction with much more complicated road network topology, in Jakarta. Table 6 shows the parameter settings of the benchmark CNN. In this study, the same parameters adopted in Alex-Net [35] were used, which were the same as in the previous work in [26]. As can be seen, this model is a typical CNN where there are three pairs of convolutions layer with 256, 128, and 64 channels, 3 × 3 kernel, max-pooling of 2 × 2, and the flattening followed by the fully connected (FC) layer. The stride of two is to capture and learn the traffic jam features, then reshaping to feed the features into the FC layer. The parameters scale of the FC layers depends on the dimension of the given input and the number of historical time step. For example, given the input 34,944 × 61 with 96 historical time steps used, then, the parameter scale of the FC layer is 703,485.

### 3.3. CapsNet-Based Traffic Jam Prediction

One drawback of the CNN-based model is that the max-pooling operator only takes the maximum value of the activation. This treatment ignores some of the information about spatial-temporal or the road network. Because all road segments in the urban transportation network are related, a small change at a certain road segment may affect other road traffic conditions. To address this issue, CapsNet, which uses road network images as inputs, can be applied. CapsNet enables the network to learn and capture the relationship of traffic jam information at certain times and spaces which presents the characteristics of the urban transportation, especially under UST condition.

Unlike CNN, CapsNet works differently where a capsule represents a group of neurons as it activates the neurons by squash nonlinearity. The network is trained using dynamic routing by agreement. The differences of CapsNet and the traditional neural network are summarized as follows:

- Either the input or output of CapsNet is a vector operation where traditional network uses a scalar;
- Before passing to the next layer, CapsNet first transforms its input into its predicted vector so-called affine transformation where traditional network does not have this mechanism;
- After weighted sum multiplies its input, squashing is used to activate the magnitude of the vector, followed by routing to determine which capsule its input should be sent to by coupling coefficient.

There are four layers in the CapsNet model, namely, convolution layer, primary capsule layer, traffic jam capsule, and reconstruction layer. In the convolution layer, a standard convolution and the filter size was applied. Used the standard nonlinearity ReLU written as:

(3) $$ReLu\;\left(s\right)=\;\left\{\begin{array}{c} s,\;\;s\;>\;0\\ 0,\;\;s\;\leq \;0 \end{array}\right..$$

As for the affine transformation of ${\hat{u}}_{j|i}$ given in Equation (4), where $w_{ij}$ is the weight learned in back propagation and $u_{i}$ is its input, and the coupling coefficient of $c_{ij}$ is calculated by the softmax function as denoting in Equation (5). Then the input layer of the parent capsule in the next layer is calculated by Equation (6). The affine transformation was done before the primary capsule layer.

(4) $${\hat{u}}_{j|i}\;=w_{ij}u_{i},$$

(5) $$c_{ij}\;=\;\frac{exp^{\left(b_{ij}\right)}}{\sum_{k}exp^{\left(b_{ik}\right)}\;},$$

(6) $$s_{j}\;=\;\underset{i}{\sum }c_{ij}{\hat{u}}_{j|i}.$$

The nonlinearity function, so-called squash, given in Equation (7) is carried out to calculate the output vector of the network, Equation (8), where the value is between 0 to 1 which is considered as a probability of the object being present. The more an object is likely to be present the higher the value is yielded, and vice-versa. In the last capsule layer, the loss is calculated for each capsule k. The loss function is denoted in Equation (9) which is similar to marginal loss function in SVM.

(7) $$Squash=\frac{{\left|\left|S_{j}\right|\right|}^{2}}{1+\;{\left|\left|S_{j}\right|\right|}^{2}}\frac{S_{j}}{\left|\left|S_{j}\right|\right|},$$

(8) $$V_{j}=Squash\left(s_{j}\right),$$

(9) $${Loss}_{k}\;={\;T}_{k}max{\left(0,{\;m}^{+}-\left|\left|V_{k}\right|\right|\right)}^{2}+\;\lambda \left(1-{\;T}_{k}\right)\;max{\left(0,\left|\left|V_{k}\right|\right|-m^{-}\right)}^{2},$$

where $T_{k}=1$ if and only if a digit classes k is present, $m^{+}=0.9$, $m^{-}$ = 0.1, λ = 0.5 down-weighting of the loss for absent digit classes, $s_{j}$= input matrix multiplication before passing to activation, ${\hat{u}}_{j|i\;}$= prediction vector, and $\;c_{ij}=$ coupling coefficient set by dynamic routing iteratively [29]. 

Equation (10) is the update function of the log probabilities.

(10) $$b_{ij}\;=\;b_{ij}\;+\;v_{j}{\hat{u}}_{j|i}.$$

Despite its capability to tackle the CNN limitation, CaspNet suffers severe computational time with respect to more complex data. Therefore, a modification of squash nonlinearity is adopted as in [30]. Thus, the nonlinearity will be more responsive to small shift and the function is given in the following Equation (11).

(11) $$Edgar_{Squash}\;=\;1-\frac{1}{\;exp^{\left|\left|S_{j}\right|\right|}}\frac{S_{j}}{\left|\left|S_{j}\right|\right|}.$$

Since the target of our model is traffic jam speed, which is one type of regression problem, then, the loss function is threated as mean squared error (MSE). Therefore, CaspNet in this research, replacing the loss function by MSE described in Equation (12).

(12) $$MSE\left({\hat{pv}}_{k},pv_{k}\right)\;=\;\sum_{k=1}^{R}{\left({\hat{pv}}_{k}-pv_{k}\right)}^{2},$$

where $pv_{k}$ is the true probabilities associated with an image and ${\hat{pv}}_{k}$ is the calculated probabilities results of the network. In this work, the typical CapsNet setting is shown in Table 7. The performance of this CapsNet is compared with CNN and the optimized version of CapsNet, which are addressed in the following section.

### 3.4. Optimized CapsNet (OCapsNet)

In fact, training CapsNet as described in the previous subsection is time consuming. Xi, E., S. Bing, and Y. Jin, attempted to validate the effectiveness over complex data such as CIFAR10 [29] by increasing the number of primary capsule, stacking more capsule layer, ensembling averaging, modifying the reconstruction loss, and customizing the squash nonlinearity. Their result suggested that ensembling averaging can improve the efficiency, and the number of convolution layers of CapsNet is suggested to be two. Stacking more capsule layer does not influence the result significantly.

Gagana, B., H.U. Athri, and S. Natarajan, investigated the changing of nonlinearity in the convolution layer of CapsNet and tested on MNIST and CIFAR10. Their result shows that Leaky ReLU and variant ReLU, e-swish outperform constantly over the ReLU [32]. Another work by Malmgren [33] surveyed and performed a comparative study of dynamic routing of the CapsNet. Wang and Liu proposed a novel optimization of dynamic routing where the loss function was seen as a clustering-like loss function [31].

On the basis of a literature study, in this work, the optimized CapsNet (called OCapsNet) particularly for traffic jam prediction was proposed. Basically, OCapsNet changes the ReLU nonlinearity as advised in the work by [32] to the leaky ReLU due to its consistency of the performance. The Leaky ReLU function can be denoted as follows:

(13) $$Leaky\;ReLu\;\left(s\right)=\;\left\{\begin{array}{c} s,\;\;if\;s\;>\;0\\ 0.01s,\;\;otherwise \end{array}\right..$$

**Figure 6:**
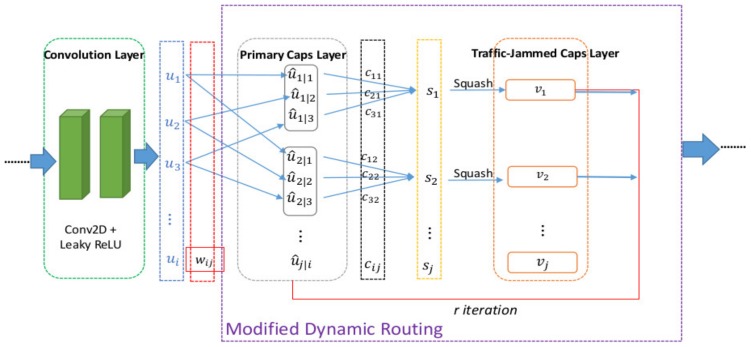
The illustration of training process of the proposed OCapsNet.

The training process in the proposed OCapsNet is illustrated in Figure 6. The format of the input data is tensor (T, R, 1). First, the input data are convolved using conv2D and Leaky ReLU as the activation function into several feature maps $u_{i}$, which is highlighted in blue line, where i is the number of feature maps. The feature maps are fed into the primary layer and transformed into the prediction vectors ${\hat{u}}_{j|i}$, where j is the number of the prediction vectors and j|i represents the connectivity of the prediction vector ${\hat{u}}_{j|i}$ and feature map $u_{i}$ through affine tranformation, with $w_{ij}$ as the weights. Then, the prediction vectors ${\hat{u}}_{j|i}$ expresses how much the primary capsule i redound to capsule j. 

Secondly, a product of ${\hat{u}}_{j|i}$ and $c_{ij}$ expresses the agreement between capsules i and capsule j is employed to get a single primary capsule i’s prediction to the class capsule at the traffic jam caps layer where $c_{ij}$ is the coupling coefficient. The higher the agreement is the more relevant the two capsules are. Thus, increasing the agreement will also be increasing the coupling coefficient.

Next, a weighted sum $s_{j}$ is computed to get the candidates for the squashing function $v_{j}$ where the squash to keep the of the output from the capsule is between 0 and 1 as a likelihood. The output from one capsule layer needs to route to the next capsule layer at particular iteration. This routing mechanism is done by dynamic routing (DR), as described in the originally proposed Algorithm 1, and $c_{ij}$ is updated by finding the dot product $v_{j}{\hat{u}}_{j|i}$ given in the Equation (5). 

**Algorithm 1** DR (Sabour et al., 2017 [29])
**Procedure** DR $\left({\hat{u}}_{j|i},\;r\;,\;l\;\right)$**For** caps i in layer l and caps j in layer $\left(l+1\right)$ : $b_{ij}\leftarrow 0$**For** r iterations **do****For** all caps i in layer $l\;:c_{i}\leftarrow softmax\;\left(b_{i}\right)$ (Equation (5))**For** all caps j in layer ($l+1)\;:s_{j}\leftarrow \;\underset{i}{\sum }c_{ij}{\hat{u}}_{j|i}$ (Equation (6))**For** all caps j in layer ($l+1)\;:v_{j}\leftarrow \;Squash\left(s_{j}\right)$ (Equation (7))**For** all caps i in layer l and caps j in layer $\left(l+1\right):\;\;b_{ij}\;\leftarrow b_{ij}+v_{j}{\hat{u}}_{j|i}$ (Equation (10))Return $v_{j}$


However, Algorithm 1 has a limitation on the cosine of the angle between two pose vectors is used to measure their agreement. The cosine saturates at 1, which makes it less sensitive to the difference between a quite good agreement and a very good agreement. In fact, the routing procedure can be seen as clustering-like such as the soft k-means algorithm, as discussed in [31]. Therefore, similar approach is carried out in our dynamic routing called modified dynamic routing (MDR) given in Algorithm 2 in this research.

**Algorithm 2** MDR (Similar with [31])
**Procedure** MDR $\left({\hat{u}}_{j|i},\;r\;,\;l\;\right)$**For** r iterations **do****For** all caps i in layer l  and caps j in layer (l+1): $\;b_{ij}=\frac{1}{\alpha }\;\langle o_{j|i},\;s_{j}\rangle \;,\;\;c_{ij}=\frac{exp^{\left(b_{ij}\right)}}{\sum_{k}exp^{\left(b_{ik}\right)}\;}$**For** all caps j in layer (l+1) : ${\hat{s}}_{j}=\sum c_{ij}o_{j|i}$ = , $s_{j}=\;{\hat{s}}_{j}/\left|\left|{\hat{s}}_{j}\right|\right|\;$**For** all caps i in layer l and caps j in layer $\left(l+1\right):\;\;w_{j}=\frac{\left|\left|\sum c_{ij}o_{j|i}\right|\right|}{1+\;max_{k}\;|\left|\sum c_{ik}o_{k|i}\right|}\;$Return $\;w_{j}s_{j}$


The objective function of the MDR algorithm can defined as follows:

(14) $$\begin{array}{c} Min\;\left(L\left(C,S\right)\;=\;-\;\underset{i}{\sum }\underset{j}{\sum }c_{ij}\;o_{j|i},\;s_{j}+\;\;\alpha \underset{i}{\sum }\underset{j}{\sum }c_{ij}\log c_{ij}\right),\\ s.t.\;\underset{j}{\sum }c_{ij}=1,\;c_{ij}>0,\;\;\|s_{j}\|\leq 1 \end{array}$$

where $o_{j|i}\;=\;\frac{1}{\left|\left|T_{ij}\right|\right|F}T_{ij}\mu_{ij}$ and $\left|\left|T_{ij}\right|\right|F$ is Frobenius norm of $T_{ij}$. α=1 .

**Figure 7:**
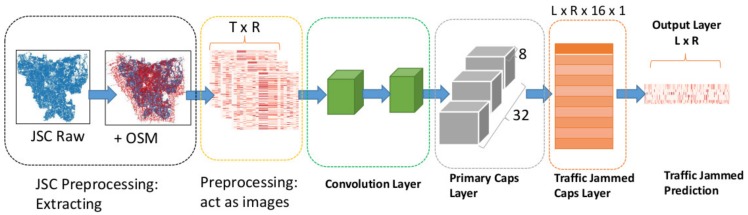
OCapsNet-based traffic jam prediction framework.

Figure 7 illustrates prediction framework of the proposed OCapsNet. Table 8 shows the network settings of OCapsNet.

In short, OCapsNet model can be summarized as follows: (a) In the first two convolution layers use Leaky ReLU rather than standard ReLU, (b) in the primary capsule layer and traffic jam capsule layer apply the modified dynamic routing, (c) investigate Edgar Squash, and (d) finally, perform the prediction at the last layer where using the MSE loss.

## 4. Experimental Results

### 4.1. Experimental Settings

In this study, two datasets were used to evaluate the prediction performance. The first dataset contained traffic jam records from 61 road segments with 15 min interval historical data and the second dataset contained 5 min interval historical data from 2972 road segment, 5 × 5 km region of West Jakarta. These two datasets represent the simple and the more complex dataset, respectively. Each dataset is divided into 70% for training while 30% for validation and testing. We then normalize the data using minimum and maximum standardization.

In this study, we used TensorFlow deep learning library running under python 3.7. and the computational resources as follows: Processor: i9-9900k 3.6 Ghz, RAM 32 Gb, and GPU NVIDIA® RTX 2080 Ti installed. ADAM optimizer [36] is being used in our implementation with initial learning rate 0.001.

In our experiments, there were four different prediction tasks. These different tasks are conducted to evaluate the performance of the proposed model on different problem complexity. Task 1, Task 2, and Task 3, considered as relatively simple prediction problems in terms of simple road topology, has the prediction tasks on the selected 61 road segments with 15 min interval by considering the previous 96, 24, and 12 observations as the features, respectively. Task 4 represents the more complex problem which has larger input images with 28 road segments in 5 × 5 km square area (larger geographic area) with 5 min interval and 12 previous observations as the features. The tasks are described as the following:

- Task 1, 15 min prediction using 24 hours (t-96) historical traffic jam data on 61 road segments;
- Task 2, 15 min prediction using 6 hours (t-24) historical traffic jam data on 61 road segments;
- Task 3, 15 min prediction using 3 hours (t-12) historical traffic jam data on 61 road segments;
- Task 4, 5 min prediction using hourly (t-12) historical traffic jam speed data on 28 roads segments in a 5 × 5 km square area.

Root mean square error (RSME), mean absolute error (MAE), and mean absolute percentage error (MAPE) are used to evaluate the prediction performance. The smaller RSME is the better result is, as well as MAE and MAPE. Let $VS_{t}$ and ${\hat{VS}}_{t}$ be the actual traffic jam speed and the predicted traffic jam speed at time t, and let M be the number of total observations, then the performance measurements can be calculated as in Equations (15)–(17).

(15) $$RMSE\;=\;\sqrt{\frac{\sum_{t=1}^{M}{\left(VS_{t}-\;{\hat{VS}}_{t}\right)}^{2}}{M}},$$

(16) $$MAE\;=\;\frac{\sum_{t=1}^{M}\left|VS_{t\;}-\;{\hat{VS}}_{t}\right|}{M},$$

(17) $$MAPE\;=\;\overset{M}{\underset{t=1}{\sum }}\frac{\left|VS_{t}-\;{\hat{VS}}_{t}\right|}{VS_{t}}\;\times \;100\%.$$

As previously mentioned, there are four prediction tasks in this experiment. In general, each task can be divided into the following two groups according to their prediction area: (1) predictions on the selected 61 road segments and (2) prediction on 5 × 5 km square area. The two groups have a different number of total observations M, i.e., 34,944 observations in group 1, and 105,120 observations in group 2.

Because different sets of activation function can produce different results [29]. Here, four sets of activation functions were tested on the proposed OCapsNet model to determine which activation should be used. Table 9 shows that evaluation results under one month of traffic data for 28 road segments on the selected 5 × 5 km square area. The best results are indicated in asterisks in Table 9. As can be seen, the Leaky ReLU, Edgar Squash, and Squash are the best for convolution layer, primary capsule layer, and traffic jam capsule layer, respectively. These setting are used for the four prediction tasks and the results are presented in the following section.

### 4.2. Experimental Results

Table 10 shows the summarized results on the prediction tasks. Please note that the reported results in Table 10 are the average of MAPE, RMSE, and MAE. The “t-96”, “t-24”, and “t-12” indicate the number of time intervals in the input image. For example, t-96 has 96 historical data points in 15 min intervals. It also means t-12 has fewer historical data points used for training.

As can be seen, CNN has better result on Task-1 which has more historical data points for training. This result is not surprising because CNN requires more data to maintain the good prediction quality. When the data point in time horizonal is fewer, CapsNet based network outperforms CNN. Particularly, OCapsNet can produce the better results than the original CapsNet.

**Figure 8:**
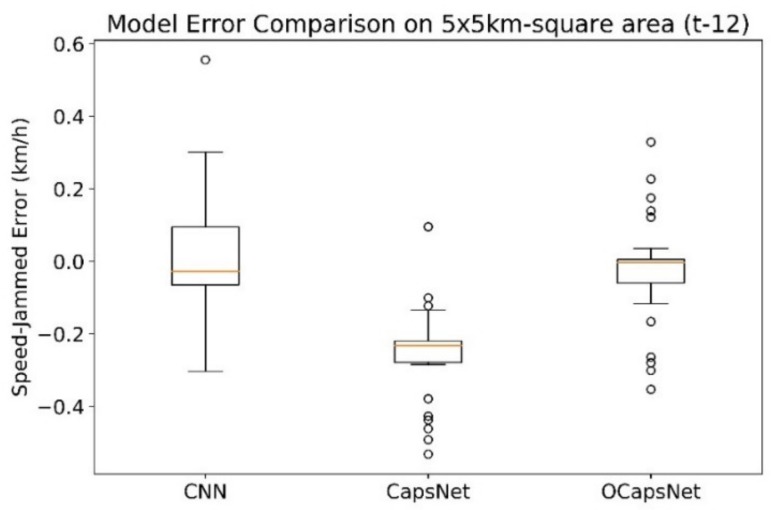
Boxplot of predicted traffic jam speed errors in km/h for Task-4.

Figure 8 plots all of prediction errors (unit is kilometer/hour) generated by CNN, CapsNet, and OCapsNet. Obviously, CNN has a much wider distribution of errors (standard deviation is 0.17) although the average of error is near zero. On the contrary, the error distributions of CapsNet and OCapsNet are much compact and their standard of the errors are 0.12, and 0.14, respectively. However, the average error of the original CapsNet is lower and shifted from zero. From Figure 8, we can also conclude the proposed OCapsNet has better prediction on traffic jam speed under UST.

## 5. Conclusions

In this work, a framework for the RNW traffic jam speed prediction under UST condition is proposed using mobile sensor data and a deep learning CapsNet-based network, called OCapsNet. In order to capture the characteristics of the spatial-temporal urban road networks, the data preprocessing on mapping the data point in traffic speed dataset with the OSM was developed. Then, the images representing the road speeds in a certain area were generated in every 5 min interval and 15 min interval. The generating images are the inputs of the deep learning network to predict the traffic jam speeds on the road network at a particular time horizon. In addition, to improve the prediction accuracy, the optimized CapsNet was proposed which performs as follows: (1) replaces the ReLU function by the Leaky ReLU function at first two convolution layers of CapsNet, (2) simplifies the dynamic routing with fewer searching loops, and (3) applies *E*dgar *S*quash function as a new activation function in Capsule layer.

The experiments were conducted to evaluate the prediction performance based on the real-world data from JSC. The performances of the original CNN and CapsNet were compared with the proposed OCapsNet. The results show that OCapsNet outperforms CNN (our benchmark) and the original CapsNet in terms of smaller MAPE, RMSE, and MAE when fewer historical data points are fed into the model. The compact error distribution also indicates that OCapsNet has better precision on prediction. 

Although the prediction result is promising, the proposed model still has a limitation of suffering severely loner time for training. How to improve the MAPE from 30% to a better result under complex road network of UST instead of on single freeway is still an open question. In addition, the dynamic routing step can be further improved by considering EM routing or other routing mechanism. In addition, it would be interesting to see if the combination of OCapsNet with LSTM can improve the model as it can fully capture the temporal interaction of the traffic road condition. Last but not least, it would be worth considering more features, such as weather, traffic event, and other traffic information represented to the channel of the image to be included for image-basis input of the deep learning model.

## Figures and Tables

**Table 1:** Examples of traffic jam speed data (Jakarta City).

-	Longitude	Latitude	Speed (km/h)	Level	Time
1	106.782318	−6.198835	2.91	3	2017-11-17 00:18:56
2	106.899734	−6.218775	1.32	4	2017-11-16 23:34:48
3	106.782875	−6.333290	3.44	3	2017-11-17 00:04:58
4	106.825172	−6.187048	2.31	3	2017-11-17 00:02:35
5	106.738623	−6.126845	3.53	3	2017-11-17 00:11:55
6	106.738625	−6.126846	3.51	3	2017-11-17 00:11:52

**Table 2:** Examples of traffic jam data with OSM data integration (Jakarta City).

-	OSM ID	Road ID	Speed (km/h)	Level	Time
1	560008540.0	9057	2.91	3	2017-11-17 00:18:56
2	28926412.0	9295	1.32	4	2017-11-16 23:34:48
3	513190960.0	9661	3.44	3	2017-11-17 00:04:58
4	513190965.0	102148	2.31	3	2017-11-17 00:02:35
5	459357692.0	192345	3.53	3	2017-11-17 00:11:55
6	459357692.0	192345	3.51	3	2017-11-17 00:11:52

**Table 3:** A list of 61 chosen roads for the experiment (Jakarta City).

-	OSM ID	Road Name
1	28809051	S. Parman Rd. (Target)
2	413471399	Other direction of S. Parman Rd.
3	566885184, 462712296	Tanjung Duren Timur Rd.
4	28225264, 560008540, 594403621	Tomang Raya Rd.
5	540317195,459357692	Tanjung Duren Raya Rd.
6	28926412, 566938121,497546919	Kyai Tapa Rd.
7	28931424	Kyai Tapa to S.Parman Turn
8	513190960, 513190965, 297913073, 28926394	Daan Mogot Rd.

**Table 4:** Examples of OSM ID and the associated Road ID (Jakarta City).

OSM ID	Road ID
28809051	[27390, 56887, 73279, 73313, 85053, 88497, 149973, 153227, 153264]
413471399	[56883, 153273]
566885184	[74322, 74330]
462712296	[74328, 115865, 116279, 116282]
28225264	[27386, 37452, 37460, 152965, 155618, 259258]

**Table 5:** Data examples for experiment.

-	Road ID	Weekday	Week Num	Time	Speed (km/h)	Level
1	307770	Wednesday	52	17:45:00	10	0
2	307770	Wednesday	52	18:00:00	6.340	1
3	307770	Wednesday	52	18:15:00	6.347	1
4	307770	Wednesday	52	18:30:00	6.856	1
5	307770	Wednesday	52	18:45:00	5.616	2

**Table 6:** Network settings of CNN based on the work in Ma et al. [26].

Layer	Names	Parameters	Nonlinearity
Input	-	-	-
Layer 1	Convolution-1	256, 3, 3	ReLU
Layer 2	Max-Pooling-1	2, 2	-
Layer 3	Convolution-2	128, 3, 3	ReLU
Layer 4	Max-Pooling-2	2, 2	-
Layer 5	Convolution-3	64, 3, 3	ReLU
Layer 6	Max-Pooling-3	2, 2	-
Layer 7	Flatten	-	-
Layer 8	Fully Connected (FC)	-	-
Output	-	-	-

**Table 7:** Network settings of CapsNet.

Layer	Name	Parameters	Nonlinearity
Input	-	-	-
Layer 1	Convol-1	32, 3, 3	ReLU
Layer 2	Convol-2	32, 3, 3	ReLU
Layer 3	Primary Capsule	(128,3,3) Capsule: 8	Squash
Layer 4	Traffic-Jammed Capsule	Capsule 16	Squash
Output	-	-	-

**Table 8:** Network settings of the proposed OCapsNet.

Layer	Name	Parameters	Nonlinearity
Input	-	-	-
Layer 1	Convol-1	256, 1, 1	Leaky ReLU
Layer 2	Convol-2	32, 3, 3	Leaky ReLU
Layer 3	Primary Capsule	(128,3,3) Capsule: 8	Edgar_Squash
Layer 4	Traffic Jam Capsule	Capsule 16	Squash
Output	-	-	-

**Table 9:** Performance with the different activation function of OCapsNet.

Convol	Primary Caps	Traffic Jam Caps	MAPE	RMSE	MAE
ReLU	Squash	Squash	13.351	0.448	0.519
Leaky ReLU	Squash	Squash	14.529	0.567	0.643
* Leaky ReLU	Edgar Squash	Squash	12.529	0.421	0.45
Leaky ReLU	Edgar Squash	Edgar Squash	13.567	0.439	0.551

* The activation function settings which are then used for the four prediction tasks.

**Table 10:** Performance comparison on four selected tasks.

Task	CNN			CapsNet			OCapsNet		
	MAPE	RMSE	MAE	MAPE	RMSE	MAE	MAPE	RMSE	MAE
Task-1 (t-96) *	**29.56**	**1.707**	**1.272**	30.46	1.749	1.307	29.74	1.709	1.274
Task-2 (t-24) *	29.42	1.702	1.282	29.28	1.701	1.266	**29.15**	**1.670**	**1.218**
Task-3 (t-12) *	29.84	1.748	1.296	33.72	1.673	1.473	**29.25**	**1.603**	**1.294**
Task-4 (t-12) **	13.99	1.166	0.592	13.15	**1.009**	0.497	**12.52**	1.049	**0.450**

* Performance comparison on 61 road segments of the selected 8 UST Roads (see Table 3). ** Performance on the selected 28 road segments in a 5 × 5 km square (see Figure 3b)
